# Gene expression profiling in human neutrophils after infection with *Acinetobacter baumannii in vitro*

**DOI:** 10.1371/journal.pone.0242674

**Published:** 2020-11-30

**Authors:** María Lázaro-Díez, Itziar Chapartegui-González, Borja Suberbiola, J. Gonzalo Ocejo-Vinyals, Marcos López-Hoyos, José Ramos-Vivas

**Affiliations:** 1 Health Research Institute Marqués de Valdecilla (IDIVAL), Santander, Spain; 2 Division of Infectious Diseases, Department of Pediatrics, UCSD, San Diego, California, United States of America; 3 Department of Microbiology and Immunology, UTMB, Galveston, Texas, United States of America; 4 Intensive Care Department, University Hospital Marqués de Valdecilla, Santander, Spain; 5 Immunology Department, University Hospital Marqués de Valdecilla, Santander, Spain; 6 Spanish Network for Research in Infectious Diseases (REIPI), ISCIII, Madrid, Spain; Universidade Nova de Lisboa, PORTUGAL

## Abstract

*Acinetobacter baumannii* is a Gram negative nosocomial pathogen that has acquired increasing worldwide notoriety due to its high antibiotic resistance range and mortality rates in hospitalized patients. Therefore, it is necessary to better understand key aspects of *A*. *baumannii* pathogenesis such as host-pathogen interactions. In this report, we analyzed both gene expression and cytokine production by human neutrophils infected with *A*. *baumannii*. Our assays reveal a proinflammatory response of neutrophils after *A*. *baumannii* infection, since intracellular transcription of effector proteins such as COX-2, transcription factors, and proinflammatory cytokines resulted significantly upregulated in neutrophils infected by *A*. *baumannii*, compared with unstimulated human neutrophils. Translation and release of CXCL-8, IL-1β and TNF-α by neutrophils was confirmed by protein quantification in culture supernatants. Results obtained in this report reinforce the importance of human neutrophils in controlling *A*. *baumannii* infections but also emphasize the proinflammatory nature of these host-pathogen interactions as a target for future immunomodulatory therapies.

## Introduction

*Acinetobacter baumannii* is a Gram negative nosocomial bacterial species that has emerged as an important pathogen causing infections such as pneumonia and bacteremia. The main problem in combating this bacteria is the high antibiotic resistance range, with some strains even presenting resistance to all the types of antimicrobials available in the clinical practice (pan-drug resistant strains) [1, 2]. Considering the importance of this pathogen, the study of its interaction with host cells becomes crucial [2].

The interaction of this bacterium with epithelial cells indicates that it has limited virulence [3]. On the other hand, the interaction of *A*. *baumannii* with cells of the immune system is starting to be better understood. At this point, neutrophils appear to be the key cell for the control of the pathogen within the human body [4]. Neutrophils, also known as polymorphonuclears (PMNs) are key cells in the innate immune response, and are considered professional phagocytes. They are, as well as macrophages, in charge of killing pathogens by phagocytosis [5]. Neutrophils are also able to eliminate pathogens by releasing cytoplasmic granules in a process known as degranulation, and by generation of neutrophil extracellular traps (NETs) [6]. In a previous work, we demonstrated the ability of human neutrophils to phagocytize *Acinetobacter* cells and to release NETs in response to infection with this bacterium [4]. Activated neutrophils can play a key role in expressing and producing proinflammatory cytokines that alert and attract other immune cells to the site of infection [5, 7] and it is important to know how these cells specifically fight *A*. *baumannii* infections.

Understanding the mechanisms by which neutrophils interact with pathogens is a prerequisite for the development of new immunomodulatory therapies to treat the infections caused by these bacteria. Therefore, in this work, we investigated the interaction of this bacterium with human neutrophils focusing on the cytokine transcription program elicited by these immune cells against *Acinetobacter*.

## Materials and methods

### Bacterial strain and growth conditions

*Acinetobacter baumannii* ATCC^®^ 19606™ strain was used in this study. Bacteria were routinely cultured on blood agar (BA) plates, brain heart infusion broth (BHIB) or Luria broth (LB) at 37°C, and frozen at -80°C with 20% glycerol.

### Neutrophil isolation from whole human blood

All studies involving human samples were in accordance with international standards for research ethics and were approved by the local institutional review board (Health Research Institute Marqués de Valdecilla). Neutrophils were isolated from whole venous blood obtained from healthy human volunteers after informed consent as previously described [4]. Briefly, the EasySep™ Direct Human Neutrophil enrichment kit (StemCell) was used, following the manufacturer’s instructions. Briefly, fifty μL of EasySep neutrophil enrichment cocktail, containing a mix of tetrameric antibody complexes produced from monoclonal antibodies directed against the cell surface antigens CD2, CD3, CD9, CD19, CD36, CD56, and magnetic beads were added per 1 mL of blood. The blood/antibody/bead solution was adjusted to a total volume of 50 mL with recommended media and placed into an Easy 50 magnet. Unbound neutrophils were pipetted into a new tube and these steps were repeated twice. Highly-pure unbound neutrophils were centrifuged and resuspended in RPMI 1640 media (Gibco) enriched with 10% fetal bovine serum (FBS) and cultured overnight (O/N) at 37°C and 5% CO_2_.

### Neutrophils infection

*Acinetobacter baumannii* was cultured O/N in 10 mL BHIB at 37°C with shaking at 175 rpm. Neutrophils were infected with bacteria at a multiplicity of infection (MOI, bacterium: eukaryotic cell ratio) of ~100:1 as previously described [4]. The numbers of colony forming units (CFUs) inoculated per well was determined by serial dilution in phosphate buffered saline (PBS), plated on BA and incubated for 24 h. The infected chamber slides were centrifuged for 4 min at 200 × *g* prior to the incubation to promote adherence of bacteria to cells and to synchronize infections. Infected cells were then incubated at 37°C with 5% CO_2_ for 2 h.

### Scanning electron microscopy

Coverslips containing infected neutrophils were fixed in ice-cold 3% glutaraldehyde for 20 min at 4°C. Samples were dehydrated, dried by the critical point method, coated with gold in a Balzers (Liechtenstein) SCD 004 sputter coater and observed with a JEOL (Tokyo, Japan) JSM-6480 LV electron microscope working at 20 kV.

### RNA extraction and cDNA synthesis

RNA from infected and unstimulated neutrophils was extracted using the Quick-RNA MiniPrep kit (Zymo Research) according to manufacturer’s instructions. RNA concentration and purity was measured using a Nanodrop™ 2000c (Thermo Fisher) and RNA was stored at -80°C for further applications. cDNA was generated from 400 ng of the total RNA using the SABiosciences's RT^2^ First Strand Kit, according to the manufacturer's protocol (Qiagen), including a DNase treatment step.

### Gene expression by q‐PCR cDNA arrays

Gene expression quantification was performed using RT^2^ Profiler™ PCR Array Human Toll-Like Receptor Signaling Pathway (PAHS-018Z). This kit profiles the expression of 84 genes involved in the Toll-Like Receptor (TLR) Signaling Pathway, including five housekeeping genes. Amplification, data acquisition, and the melting curve were carried out by means of the CFX‐Manager software (BioRad). The PCR cycling program was set as follows: step 1: 95°C for 10 min, step 2: 95°C for 15 sec, and followed by 60°C for 1 min repeated for 40 cycles. The threshold cycle (*C*_t_) and melting curve of each gene were automatically established and recorded by the software.

For gene expression experiments results were analyzed using the PCR Array Data Analysis Web Portal (Qiagen) as previously described [8, 9]. Briefly, the delta *C*_t_ (ΔC_t_) method was used for PCR array data analysis. The normalized (ΔC_t_) for each gene of interest (GOI) was calculated by subtracting the average *C*t of the five housekeeping genes from the *C*t of each GOI. Next, the double delta *C*t (ΔΔC_t_) for each GOI was calculated by deducting the average ΔC_t_ of GOI in the sham group from the ΔC_t_ of each GOI. The fold‐change of each GOI compared with the sham group was calculated as equation image.

### Cytokines quantification

IL‐8, IL-1β and TNF-α levels in supernatants of neutrophil cultures infected with *A*. *baumannii* were quantified by using the Luminex platform with a magnetic system (Milliplex MAP Human High Sensitivity Bead Panel, EMD Millipore Corporation, Billerica MA). Briefly, after *A*. *baumannii* infections, the culture medium was collected at different time points and filtered (0.22 μm), and 100 μL of supernatant were used for cytokine quantification according to the manufacturer's instructions. Data are presented from three independent experiments.

### Data and statistical analysis

For gene expression, *C*t data were uploaded into the data analysis template on the manufacturer's website and *p*-values were calculated based on a Student's *t*‐test of the replicate values for each gene in the control and infected groups. Fold‐regulation represents fold‐change results in a biologically meaningful way. A fold‐change value greater than 1 indicates positive or an overexpression, and the fold‐regulation is equal to the fold‐change. Fold‐change values less than 1 indicate negative or downregulation, and the fold‐regulation is the negative inverse of the fold‐change. For cytokine measurements, supernatants from 3 independent experiments were collected, and data were represented using GraphPad Prism 5.0. Cytokines produced by neutrophils infected with ATCC® 19606™ and unstimulated neutrophils were compared using *t*-test. *P*-values less than 0.05 were considered significant. Data are presented as mean ± SE (standard error) of three independent experiments.

## Results

### Microscopy

**Fig 1:**
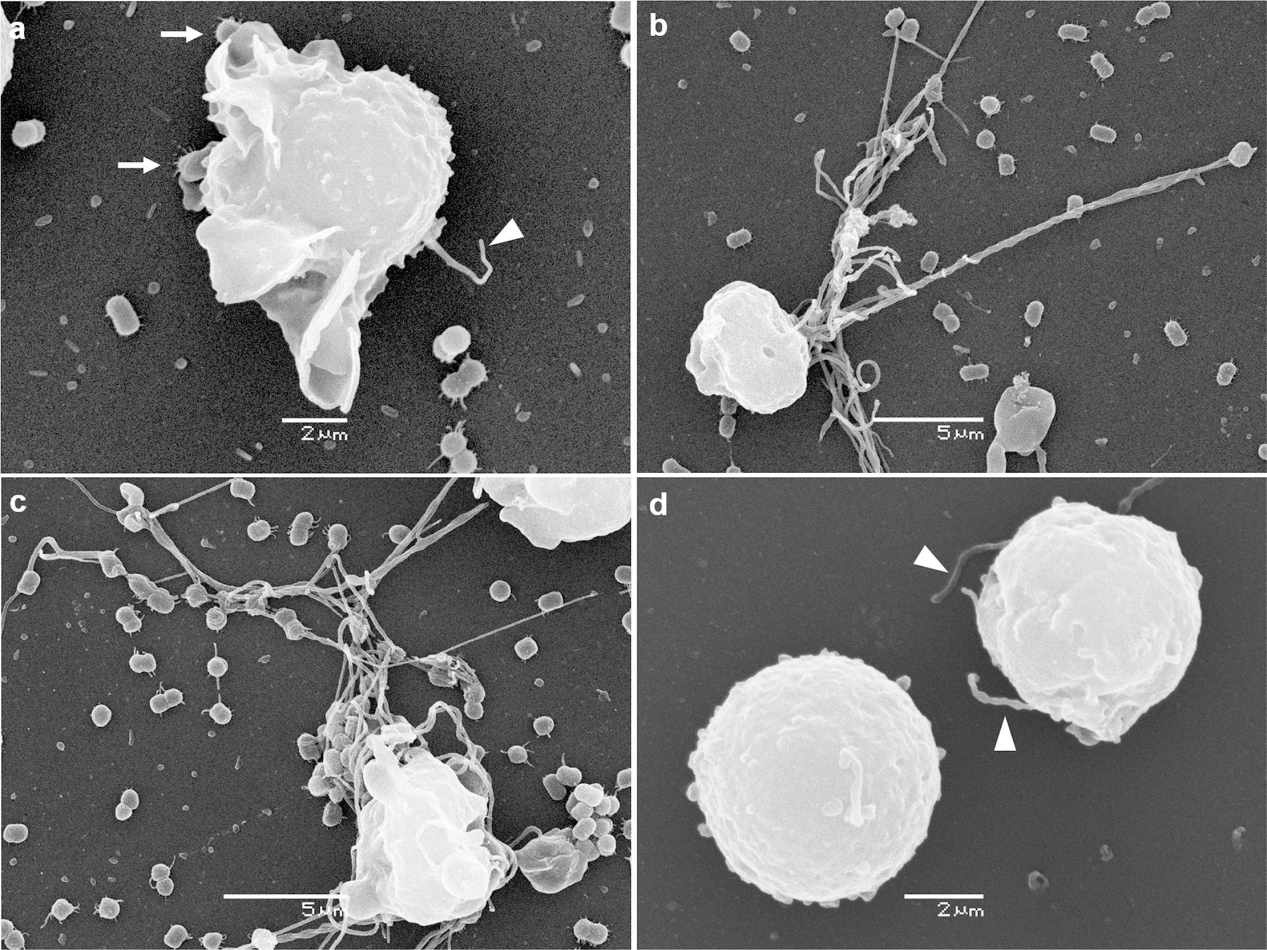
Capture of *Acinetobacter* by human neutrophils through lamellipodia and filopodia. Pictures show SEM microphotographs of control or infected neutrophils (2 h). Short lamellipodia (arrows in **a**) or large filopodia (**b**, **c**) were observed surrounding bacteria. Arrowheads in **a** and **d** (unstimulated neutrophils) show short filopodia. Results are representative of observations made from at least three independent experiments. Micrographs were originally captured at ×6500 (**a**), ×4500 (**b**), ×5000 (**c**), ×8000 (**d**) magnification.

SEM results are shown in Fig 1. Human neutrophils are round cells that remain semi-attached to the plastic surface (Fig 1A), becoming flatter with phagocytic activity in presence of bacteria (Fig 1B). The transition to active phagocytosis is sudden and progresses to complete engulfment of the bacteria. Also, the extension of the cell-bacteria contact area is followed by the emergence of pseudopods or filopodia. These large filopodia (>50 μm) seem to bind and/or retain bacteria (Fig 1C and 1D).

### Gene expression

Results from gene expression analysis are shown in Table 1. In neutrophils infected with *A*. *baumannii* ATCC^®^ 19606™ for 2 h, a total of 41 out 84 genes were overexpressed and 2 of them were downregulated, compared with unstimulated neutrophils as control (Table 1). Among all of them, the difference in expression was statically significant (*p*<0.05) in 6 of these genes.

**Table 1:** TLR-4 immune-related genes in neutrophils whose transcript levels exhibited twofold or greater modulation after infection with *A*. *baumannii* at 2h.

GENE SYMBOL	DESCRIPTION	FOLD REGULATION
**BTK**	Bruton agammaglobulinemia tyrosine kinase	20.16
**CHUK**	Conserved helix-loop-helix ubiquitous kinase	8
**CSF2**	Colony stimulating factor 2 (granulocyte-macrophage)	4
**CSF3**	Colony stimulating factor 3 (granulocyte)	12.7
**CXCL10**	Chemokine (C-X-C motif) ligand 10	3.17
**ECSIT**	ECSIT homolog (Drosophila)	20.16
**EIF2AK2**	Eukaryotic translation initiation factor 2-alpha kinase 2	2
**ELK1**	ELK1, member of ETS oncogene family	5.04
**FOS**	FBJ murine osteosarcoma viral oncogene homolog	10.08
**IL10**	Interleukin 10	2
**IL1A**	Interleukin 1, alpha	32
**IL1B**	Interleukin 1, beta	32^a^
**IL6**	Interleukin 6 (interferon, beta 2)	20.16
**CXCL8**	Interleukin 8	16^a^
**IRAK1**	Interleukin-1 receptor-associated kinase	5.04
**IRAK2**	Interleukin-2 receptor-associated kinase	3.17^a^
**IRAK4**	Interleukin-4 receptor-associated kinase	3.17
**IRF3**	Interferon regulatory factor 3	2
**LTA**	Lymphotoxin alpha (TNF superfamily, member 1)	3.17
**LY86**	Lymphocyte antigen 86	16
**MAP2K3**	Mitogen-activated protein kinase kinase 3	5.04
**MAP4K4**	Mitogen-activated protein kinase kinase 4	3.17
**MAPK8**	Mitogen-activated protein kinase kinase 8	2.52
**MAPK8IP3**	Mitogen-activated protein kinase 8 interacting protein 3	5.04
**NFKB1**	Nuclear factor of kappa light polypeptide gene enhancer in B-cells 1	8^a^
**NFKB2**	Nuclear factor of kappa light polypeptide gene enhancer in B-cells 2 (p49/p100)	4
**NFKBIA**	Nuclear factor of kappa light polypeptide gene enhancer in B-cells inhibitor, alpha	5.04
**NFKBIL1**	Nuclear factor of kappa light polypeptide gene enhancer in B-cells inhibitor-like 1	3.17
**NR2C2**	Nuclear receptor subfamily 2, group C, member 2	2
**PPARA**	Peroxisome proliferator-activated receptor alpha	8
**PTGS2**	Prostaglandin-endoperoxide synthase 2 (prostaglandin G/H synthase and cyclooxygenase)	128^a^
**REL**	V-rel reticuloendotheliosis viral oncogene homolog (avian)	4
**RIPK2**	Receptor-interacting serine-threonine kinase 2	3.17
**SIGIRR**	Single immunoglobulin and toll-interleukin 1 receptor (TIR) domain	5.04
**TAB1**	TGF-beta activated kinase 1/MAP3K7 binding protein 1	4
**TBK1**	TANK-binding kinase 1	3.17
**TICAM1**	Toll-like receptor adaptor molecule 1	6.35
**TLR4**	Toll-like receptor 4	3.17
**TLR6**	Toll-like receptor 6	6.35
**TNF**	Tumor necrosis factor	5.04^a^
**TNFRSF1A**	Tumor necrosis factor receptor superfamily, member 1A	6.35
**TOLLIP**	Toll interacting protein	4
**TRAF6**	TNF receptor-associated factor 6	5.04
**ACTB**	Actin, beta	10.08
**CD14**	CD14 molecule	-2.52
**PELI1**	Pellino homolog 1 (Drosophila)	-3.17

^a^Denotes genes that were upregulated or downregulated in infected neutrophils with respect to the controls with a fold regulation ≥2 and a *p*-value <0.05 from three independent experiments.

### Cytokines quantification

**Fig 2:**
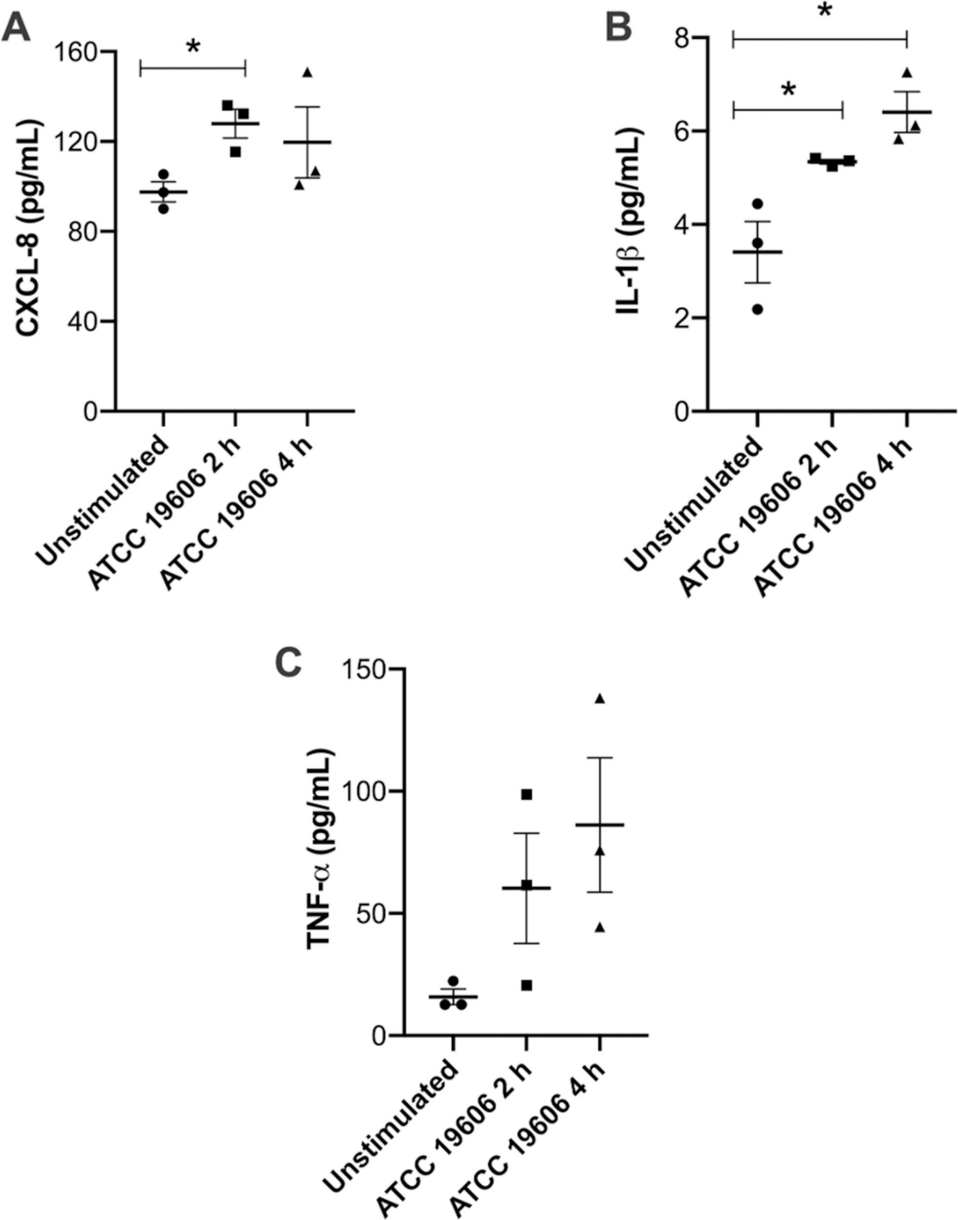
**CXCL-8 (A), IL-1β (B), and TNF-α (C) concentrations in culture supernatants from neutrophils unstimulated and stimulated with *A*. *baumannii* ATCC^®^ 19606™ for 2 and 4 h.** Bars represent the standard error (SE) from 3 independent experiments measured in duplicate; asterisks denote statistically significant differences (*p*<0.05).

Since CXCL-8, IL-1β and TNF-α resulted as statistically significant overexpressed cytokines after 2h post infection, we analyzed their concentration at a protein level in supernatants of neutrophils infected with *A*. *baumannii* ATCC^®^ 19606™ also 4 h after infection (Fig 2). We found an increase in CXCL-8 at both time points of infection (Fig 2A). We also measured an increase of IL-1β (Fig 2B) levels secreted at 4 h of infection, and the same pattern was repeated for TNF-α (Fig 2C).

## Discussion

The research on host-bacteria interactions is an evolving field that pursues to discover new treatments against infectious diseases. One of the strategies is to modulate the disproportionate response that occurs when immune cells interact with some pathogenic bacteria and produce a series of cytokines that lead to an exacerbated inflammatory response that may be more difficult to control than the infection itself. This is sepsis, and one of the key cells involved in sepsis is the neutrophil [10–12]. One bacterium that releases high amounts of lipopolysaccharide (LPS) during infection is *A*. *baumannii* [13]. Therefore, it is important to know the response of neutrophils against bacteria or against bacterial products such as LPS. *Acinetobacter* does not have special factors to be virulent, it does not have important toxins, or mobility, and lacks strong adherence to human epithelial cells *in vitro* [3, 13], but it is quickly phagocytized by neutrophils [4]. Logically, this phagocytosis stimulates neutrophils to generate an immune response as antigen presenting cells [14]. Several *in vivo* (mice) studies have demonstrated the importance of neutrophils in combating *A*. *baumannii* [15]. On the other hand, studies in murine [16, 17] and human macrophages [18] showed that *A*. *baumannii* LPS is a key activator of the TLR-4. Thus, it is important to know how human neutrophils respond to this pathogen through the activation of TLRs. In this report, we demonstrated, at a transcriptional level, the activation of human neutrophils in response to this infection, which could correspond to a significant expression of TLR4. RNA extraction in neutrophils is especially concerning. Firstly, neutrophils hold 10–20 times less RNA than other leukocytes [19]. For this reason, the technique of neutrophil isolation from peripheral blood should guarantee maximal purity of these cells. In this report, we used a negative selection commercial method that ensures maximum purity in the extraction. Another key aspect in gene expression analysis in neutrophils is the selection of housekeeping genes. Due to the fact that the expression of housekeeping genes commonly used in other cell types is not always stable in neutrophils, it is necessary to choose housekeeping genes that are stable among the different experimental conditions [20]. In this case, we used the housekeeping genes β-2-microglobuline (B2M) and Glyceraldehyde 3-phosphate dehydrogenase (GAPDH) in whose *C*t did not suffer variations in the different conditions tested.

Interestingly, the gene that appears highly overexpressed among all of the genes tested was Prostaglandin-endoperoxide synthase 2. This gene codifies the expression of cyclooxygenase 2 (COX-2), a peroxidase. Overexpression of this gene in human neutrophils has already been described in response to other pathogenic bacteria such as *Helicobacter pylori* [21] but its role in controlling bacterial infections is poorly understood.

Other significantly overexpressed proteins, such as NFκB and IRAK-2 are important in cell activation; NFκB especially enhances the expression of multiple proinflammatory genes and it is known to be overexpressed in response to the TLR-4 activation pathway [22].

Furthermore, in this work, we found several proinflammatory cytokine genes overexpressed in response to *A*. *baumannii* infection such as those for the cytokines IL-6, IL-1β and CXCL-8. The genes coding for IL-1β and CXCL-8 were significantly overexpressed. We also confirmed the production of their products by ELISA, indicating that both transcription and translation of these proteins is stimulated by *A*. *baumannii* infection. All these overexpressed genes are found to be downstream of a TLR-4 inflammatory response. Then, we proposed an activation of human primary neutrophils in response to *A*. *baumannii* based on the TLR-4 cellular pathway. Using the negative selection enrichment kit to isolate neutrophils directly from whole blood will carry some contaminating cells. When we carry out this protocol, it is very difficult to isolate cells other than polymorphonuclear (PMN) cells [4]; however, this possibility exists, and could explain for example the low level of IL-10 mRNA expression. In this way, although we do not know how the bacterium can induce neutrophil gene expression *in vivo*, the inflammatory environment might be influenced by many other cells. Despite this possibility, our results correlate well with *in vivo* previous reports, where they described the importance of early cytokines expressed by neutrophils in a mouse model [15].

Interestingly, other authors observed an increase in the expression of several pro-inflammatory cytokines such as IL-1β, TNF-α or IL-6 in human polymorphonuclear leukocytes stimulated with LPS from several bacterial pathogens. However they did not find changes in CXCL-8 as we have shown in this report [21]. To our best knowledge, this is the first report where whole *A*. *baumannii* bacterium has been used to analyze the gene expression of primary human neutrophils and, therefore, these differences could be due to the use of whole bacteria as opposed to LPS alone. The mechanisms of disease caused by *A*. *baumannii* were recently reviewed [2, 13]. Our microscopy assays showed that *A*. *baumannii* is captured through lamellipodia and filopodia, reinforcing the idea that this pathogen is easily captured by human neutrophils. Phagocytosis can be measured using different assays. In a previous manuscript, we have used other techniques (up to 4 different assays) and we believe appropriate to show here at least one of them, where phagocytosis is clearly appreciated. These pictures reinforce the message that this pathogen is easily phagocytosed by this cell type. Taken together with previous studies [7, 15], these results highlight the importance of neutrophils in *A*. *baumannii* pathogenesis, not only in the elimination of bacteria by phagocytosis and NETs production, but also for inflammatory cytokine synthesis and release that allows the attraction other innate and adaptive cells to the site of infection. However, an exacerbated activation of these cells in response to the infection can be considered a potential risk for developing sepsis [23]. Other recent studies further demonstrated that interruption or reversal of the impaired migration and antimicrobial function of neutrophils improves the outcome of sepsis in animal models [24]. Our work reinforces the idea that human neutrophils control *A*. *baumannii* infections, and also emphasizes that the proinflammatory nature of these host-pathogen interactions via the TLR4 pathway could be a target for future immunomodulatory therapies.
